# Reply to Cotrim, N.; Cotrim, C. Is It Wise to Forget Exercise Stress Echocardiography in the Study of Chest Pain in Children? Comment on “Huang, S.-W.; Liu, Y.-K. Pediatric Chest Pain: A Review of Diagnostic Tools in the Pediatric Emergency Department. *Diagnostics* 2024, *14*, 526”

**DOI:** 10.3390/diagnostics15091110

**Published:** 2025-04-27

**Authors:** Szu-Wei Huang, Ying-Kuo Liu

**Affiliations:** 1 Emergency Department, Wan Fang Hospital, Taipei Medical University, Taipei 11695, Taiwan; sean04091987@gmail.com; 2 Department of Pediatrics, Wan Fang Hospital, Taipei Medical University, Taipei 11695, Taiwan

We sincerely thank Nuno Cotrim and Carlos Cotrim for their valuable comments on our article [1]. We humbly accept your insights and hope that our response meets your expectations.

As referenced in your outstanding article [2], “Clinical Application of Exercise Stress Echocardiography in an Outpatient Pediatric Population”, a total of 309 consecutive children with a mean age of 14.1 ± 2.6 years (range 6–17 years) underwent treadmill-based exercise stress echocardiography (ESE) starting in 2002. These patients were divided into two groups. Group I consisted of 258 children, including 237 with exercise-related symptoms such as chest pain, fatigue, lipothymia/syncope, or one case of aborted sudden death; 15 with electrocardiogram (ECG) abnormalities; and 6 with a positive ECG stress test showing ST changes. Group II included 10 asymptomatic children whose parents requested routine screening, 11 with symptoms unrelated to exercise, 12 with a family history of sudden death, and 17 with known cardiac pathologies, including hypertrophic cardiomyopathy (*n* = 10), aortic coarctation (*n* = 2), and other conditions such as Cortriatriatum sinister, pulmonary stenosis, subaortic stenosis, bicuspid aortic valve, left ventricular hypertrophy related to arterial hypertension, and aortic switch operations. The study findings revealed stress-induced regional wall motion abnormalities in two children. Additionally, significant transvalvular or intraventricular gradients (IVGs) exceeding 30 mmHg were detected in 101 out of the 258 children in Group I (39%), with the odds ratio (OR) of ESE reproducing symptoms in children with IVG compared to those without IVG being 8.22 (95% CI: 4.84–13.99, *p* < 0.001). In Group II, the 10 children diagnosed with hypertrophic cardiomyopathy were non-obstructive before exercise, and 4 developed intraventricular obstruction during exercise.

This study demonstrates that many adolescents with exercise-induced symptoms exhibit significant positive findings on ESE (39%). This highlights the indispensable role of ESE in comprehensive cardiac evaluations in adolescents.

Exercise stress testing plays a crucial role in cardiac function assessment. El Assaad et al. [3], in their article “Value of Exercise Stress Echocardiography in Children with Hypertrophic Cardiomyopathy”, noted that ESE can be performed safely and serves as an effective tool for identifying low-risk patients regarding cardiac outcomes in children with hypertrophic cardiomyopathy. Similarly, Dasgupta et al. [4], in their article “The Utility of Combined Cardiopulmonary Exercise Stress Testing in the Evaluation of Pediatric Patients with Chest Pain”, emphasized the widespread use of exercise stress testing (EST) in determining prognosis in patients with suspected or established coronary disease. Soham et al. also found that 1% of patients previously undiagnosed with heart disease had an abnormal stress test and incidental anomalies on echocardiography. These studies emphasize the importance of using various exercise stress tests in cardiac function evaluation.

In our article [5], we admittedly provided limited coverage of certain cardiac diagnostic tools, including exercise stress echocardiography, the treadmill stress test, magnetic resonance imaging, cardiac computed tomography, cardiac catheterization, electrophysiological study, etc. These diagnostic tools provide more comprehensive and accurate evaluations and help to avoid missed diagnoses. Each tool also has different reliability and validity for specific cardiac conditions. However, due to the limitations of our retrospective review article, many newer and more accurate techniques are continuously being developed, which may provide more precise and in-depth insights into the causes of pediatric chest pain in the future.

Dr. Nuno Cotrim and Prof. Carlos Cotrim mentioned the following in “Clinical Application of Exercise Stress Echocardiography in an Outpatient Pediatric Population?”:

“Our analysis of this group of children informs us that if we do not search for intraventricular gradients, we will not find them, losing information that may give us a clinical solution for this health problem in children”.

Furthermore, their study found that among children or adolescents who experienced exercise-induced chest pain, 39% exhibited significant intraventricular gradients (IVGs > 30 mmHg) after undergoing treadmill exercise stress echocardiography (ESE). This study demonstrates that treadmill ESE holds significant diagnostic value in evaluating pediatric chest pain and should not be overlooked.

We greatly admire Dr. Nuno Cotrim and Prof. Carlos Cotrim for their advocacy and promotion of ESE in emergency settings. With advancements in medical technology and the increased accessibility of ESE, this examination should be considered in the evaluation of pediatric chest pain cases in emergency departments.

Finally, we once again express our gratitude to Nuno Cotrim and Carlos Cotrim for their thoughtful comments, which have deepened and broadened the scope of our discussion.
